# A controlled vocabulary for pathway entities and events

**DOI:** 10.1093/database/bau060

**Published:** 2014-06-20

**Authors:** Steve Jupe, Bijay Jassal, Mark Williams, Guanming Wu

**Affiliations:** ^1^European Bioinformatics Institute (EMBL-EBI), European Molecular Biology Laboratory, Wellcome Trust Genome Campus, Hinxton, Cambridge CB10 1SD, UK; ^2^Ontario Institute for Cancer Research, Toronto, ON M5G0A3, Canada

## Abstract

Entities involved in pathways and the events they participate in require descriptive and unambiguous names that are often not available in the literature or elsewhere. Reactome is a manually curated open-source resource of human pathways. It is accessible via a website, available as downloads in standard reusable formats and via Representational State Transfer (REST)-ful and Simple Object Access Protocol (SOAP) application programming interfaces (APIs). We have devised a controlled vocabulary (CV) that creates concise, unambiguous and unique names for reactions (pathway events) and all the molecular entities they involve. The CV could be reapplied in any situation where names are used for pathway entities and events. Adoption of this CV would significantly improve naming consistency and readability, with consequent benefits for searching and data mining within and between databases.

**Database URL:** http://www.reactome.org

## Controlled names introduction

Protein sequence databases have existed for decades (1), but there is no universal authoritative source of names for proteins. Neither is there an agreed vocabulary that encompasses cleaved peptide fragments or co- and post-translationally modified forms. This presents a problem for anyone who requires an unambiguous name for a translation product and the peptides derived from it. Such modifications are often central to a protein’s function. Annotating and naming them in a way that is brief, yet distinguishes them accurately, while respecting standards in use within the scientific community, is highly desirable. Reactome, like many other databases, uses names from the scientific literature or UniProt descriptive names (2). However, it is widely recognized that names used in scientific literature are often ambiguous (3) and seldom universally applied (4). Names used in literature may be unique in the published context but ambiguous when used in a database. Often, the modified peptides, complexes and sets that are detailed in Reactome pathways are mechanistic intermediates, which have no names in the literature. The difficulties of defining names for peptides and their fragments or modified forms are amplified when they associate with other biological molecules in the cell to form complexes, or when an annotator organizes groups of molecules with shared features into sets. Consistent and informative names are equally important for the pathway events that involve these molecules or groups of molecules. Users frequently wish to identify events that belong to a distinct functional category, such as binding or phosphorylation. It may improve their understanding if the name of the event includes a term that categorizes it. Reactome associates Gene Ontology (GO) molecular function terms (5) with catalytic events but the function terms are not necessarily incorporated into reaction names. Like most other pathway databases, there is no controlled annotation that can be used to identify functional categories of reaction. Clearly, it is desirable to devise a means (or perhaps several) to provide names for these events that would indicate categories recognizable to biologists and provide a means to identify event categories using text-based querying.

Diligent curators can manually create unique names for all molecular entities and events but this has a number of difficulties. It becomes increasingly difficult to coin unique names as the database grows in size. In a pathway context, many events and the molecular objects that are involved are unnamed in the literature. Names that are created by the expert author and curator on an as-needed per-pathway basis may not be recognizable or interpretable by other biologists. Considering and defining new names while ensuring that they are unique can be a time-consuming process. Even experienced curators can make mistakes and introduce non-unique names or typographical errors. Arbitrary internal identifiers are reliably unique but essentially devoid of meaning outside the database context. It is preferable to use a controlled systematic system of names that are based on familiar biological names and terms, minimize ambiguity and support data mining.

To address these problems, we have defined a controlled vocabulary (CV) that can be used for all human pathway events (reactions) and the molecular entities that participate in these events. This benefits Reactome because it simplifies the time-consuming process of defining and maintaining unambiguous names in a consistent manner. It has benefits for anyone who uses our data by providing reliable systematic names that incorporate familiar names and existing standards to facilitate text-based searches that are currently not possible. The CV was created for use within the Reactome database but is sufficiently generic that it could form the basis of a standard nomenclature for the biological molecules and events that constitute any biological network or pathway.

## Peptide CV names

Peptides are the backbone of most pathway processes and by far the most abundant class of pathway object in Reactome. Within the Reactome data model, a peptide is represented as an ‘Entity With Accessioned Sequence’ (EWAS), which combines a reference to an external peptide database that preferentially is UniProt, and a GO cellular compartment term. All EWASes in Reactome have been renamed by a scripted process; previous names were retained as aliases. New EWAS instances are named manually and the new names verified as part of Reactome’s quarterly release process. Some EWASes are currently exempt from the CV process to prevent name duplications that would otherwise occur because the EWAS represents an uncertain modification or a modified state that is not currently covered by the CV. The Exemptions section below provides more details.

### Explanation of peptide CV names

#### Gene symbol core

The peptide CV name is constructed around a ‘core’ Human Gene Organization (HUGO) Gene Nomenclature Committee (HGNC) approved gene symbol. The HGNC is the only worldwide authority that assigns standardized nomenclature to human genes, and as such is widely recognized as the authoritative source of names for genes (6). No such similar body exists for protein names; consequently, HGNC is the only practical source of short unambiguous names that can be used to name the translated products of those genes. Reactome obtains HGNC gene symbols indirectly from UniProt, an attribute associated with every peptide EWAS.

#### Peptide coordinates suffix

Reactome frequently contains several peptides derived by the processing of a single translated peptide. These peptides are represented as separate EWAS instances having a common referenceEntity attribute that associates all with the same initial translation product and gene symbol obtained from UniProt. To generate unique names for these multiple peptides, the start and end coordinates of the EWAS are compared with UniProt's ‘Chain’ feature, part of an annotation group in UniProt called Sequence annotation Features. This feature is used to indicate the ‘default’ peptide. If the start and end coordinates of the EWAS agree with those of the Chain feature, the coordinates are deemed unnecessary and not added to the gene symbol. If either Reactome coordinate does not agree with the UniProt chain, both coordinates, separated by a hyphen, are added in round brackets as a suffix to the gene symbol. Uncertain peptide coordinates are represented as ‘?’ symbol. If the UniProt record has no chain feature or more than one chain feature, start-end coordinates are added to every EWAS derived from it.

This combination of gene symbol and coordinates is usually sufficient to generate a unique name, with the following exception. Peptide fragments generated from the cleavage of a peptide that has uncertain start or stop coordinates, or peptides that are cleaved at more than one uncertain site, can have duplicated names. When this occurs, the resulting peptides are named by following the CV naming process, with an additional manual step that creates a unique name. An indexed suffix could be used to uniquely extend the peptide CV name in these unusual circumstances, but this occurrence is sufficiently rare that the manual step is manageable and allows the name to be tailored to the exact circumstances.

##### Examples

Caspase-9 precursor, with peptide coordinates start:1 end:416 is named CASP9. The large and small subunits of caspase-9 are respectively named CASP9(1–315) and CASP9(316–416).

An N-terminal fragment of Aggrecan, generated by a second cleavage at an unknown site after removal of a leader polypeptide, would be named ACAN(17–?).

Note that for consistency Reactome peptide coordinates always refer to the UniProt Chain, even when the literature convention is to number a cleaved fragment following the removal of a signal peptide or initiating methionine.

#### Post-translational modification prefixes

Post-translational modifications (PTMs) are represented by adding a prefix and hyphen to the gene symbol. Reactome defines PTMs with hasModifiedResidue annotations. These use terms obtained from the Protein Standards Initiative modifications (PSI-MOD) ontology (7), which can be searched via the Ontology Lookup Service (http://www.ebi.ac.uk/ontology-lookup/init.do). PSI-MOD terms are cross-referenced to the RESID database (8). The correct prefix is determined by using the PSI-MOD ID to select a prefix from a cross-reference table (http://wiki.reactome.org/index.php/Systematic_Peptide_Names). Some infrequently used PTM types use the unabbreviated PSI-MOD term as a prefix. In principle, this system can be expanded to include any PSI-MOD term when its usage becomes sufficiently common to make an abbreviation desirable.

Reactome annotation specifies the coordinate position of PTMs when this is known. In the peptide CV name, coordinate position may be included after the prefix that defines the PTM type. Reactome has decided not to include the PTM coordinate except for di-lysine, tri-lysine and arginine methylation, lysine acetylation, ubiquitination and most frequently, phosphorylation. Coordinates should always be included for these PTM types to avoid name duplications. PTM prefixes for phosphorylation use a letter to distinguish between the phosphorylation subtypes of serine (S), threonine (T), tyrosine (Y) and unknown type. Multiple phosphorylations of the same peptide are ordered by their coordinate positions.

Multiple occurrences of any PTM that does not include the coordinate position should precede the coordinate by *n*x where *n* is the number of occurrences.

##### Examples of phosphorylation prefixes

**p-Y139-DAPP1** is DAPP1 phosphorylated on tyrosine-139.
**p-Y150,S343,T346-WASF2** is WASF2 phosphorylated on tyrosine-150, serine-343 and threonine-346. Note that phosphorylations are ordered by coordinate.
**p-Y55,S112,S121,Y227-SPRY2**—note that the ordering is by coordinate, not grouped by phosphorylation subtype.
**p-Y-GAB2** is GAB2 phosphorylated on a tyrosine; the coordinate position of this tyrosine is unknown.
**p-GLI3** is GLI3 phosphorylated; both the phosphorylation subtype and position are unknown.

When combinations of phosphorylation and another PTM occur, the phosphorylations are represented after everything else:

**2xPalmC-MyrG-p-S1177-NOS3(2-1203)** is NOS3 fragment 2-1203 with two palmitoylated cysteines, one myristoylated glycine and a phosphorylation on serine-1177.

#### Exemptions to peptide CV naming

At present, some Reactome peptides are exempted from CV naming. The diverse reasons for this are detailed below.

Exemptions are made when:

1. The peptide has a disease-associated mutation. We are currently developing an extension to the CV, which will cover modifications to peptides because of mutation and other events that contribute to disease processes.
2. The referenceEntity is a specific isoform. In Reactome this is determined when the EWAS attribute referenceEntity is a ReferenceIsoform with variantIdentifier greater than one. This avoids using potentially misleading UniProt coordinates for isoforms that are not represented as the ‘canonical’ isoform.
3. The peptide has a modification that is not a simple residue modification. Reactome has a number of less common residue modification classes, e.g. internal peptide cross-links that are not covered by the CV.
4. Peptides that have an active conformation, but a covalent structure that is unchanged from that of an inactive precursor. In Reactome, this distinction is made by prefixing the peptide CV name with the word ‘Active’.
5. The UniProt record has more than one or no Chain feature. This requires a manual examination of the UniProt record so that the appropriate chain is used to select peptide coordinates. In most cases where multiple chains exist in UniProt, a precursor peptide is cleaved into fragments represented as additional chains. Reactome curators will select the precursor and refer to its coordinates.
6. Human Leukocyte Antigen (HLA) genes can have thousands of alleles. An established nomenclature exists for this group of genes (http://hla.alleles.org/nomenclature/index.html), consequently they are not covered by the CV.

Peptides may be manually exempted from the CV renaming process:

1. If the CV process creates duplicated names. As described above, this can occur when peptides with uncertain start, stop or cleavage site coordinates are subsequently cleaved. This can result in multiple fragments with uncertain start–stop coordinates and consequently the same CV name. When this occurs the fragments are manually named, using the CV naming process with an additional manual step to ensure uniqueness.

A spreadsheet listing all current exemptions is available here (https://docs.google.com/spreadsheet/ccc?key=0AhLs PL1kOR– dE90UGlwTFdxWDVoT1VvTnFwNFhp OVE# gid=0).

## Small molecule (chemical) CV names

Reactome represents >1400 small molecules, cross-referenced to the Chemical Entities of Biological Interest (ChEBI) database (9). In many cases, Reactome uses the ChEBI name or an existing ChEBI synonym.

We have constructed a list of abbreviations to use in preference to the full ChEBI name for many commonly occurring small molecules. In scientific literature, it is common to see small molecules represented by an abbreviation; ChEBI includes many of these as synonyms. A standardized list of short familiar abbreviations are ideal for use as the CV names for commonly occurring small molecules.

It was often possible to identify a suitable abbreviation from ChEBI, Kyoto Encyclopedia of Genes and Genomes (KEGG) Compounds (http://www.genome.jp/kegg/compound/) (10) or PubChem (http://pubchem.ncbi.nlm.nih.gov/) (11). When a suitable name could not be found in these databases, we referred to scientific literature and an acronym website (http://www.allacronyms.com/) to suggest abbreviations from the categories medical or science. In the absence of any existing abbreviation, we created our own, e.g. 1D-myo-Inositol 1,3,4,5,6-pentakisphosphate was abbreviated to I(1, 3, 4, 5, 6)P5, and 4-(4-(dimethylamino)styryl)-*N*-methylpyridinium was abbreviated to 4-Di-2-ASP.

A full list of the abbreviations used in Reactome is available on our Wiki pages (http://wiki.reactome.org/index.php/Small_Molecule_Abbreviations).

**Figure 1.**
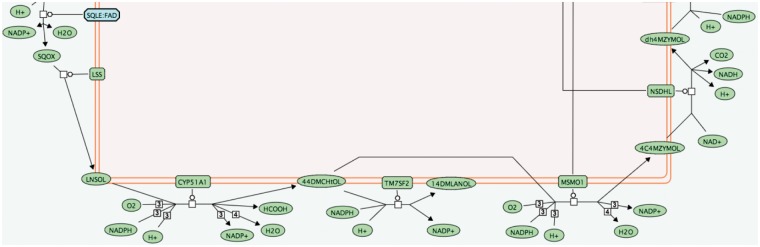
Pathway diagram segment using abbreviated small molecule names.

**Figure 2.**
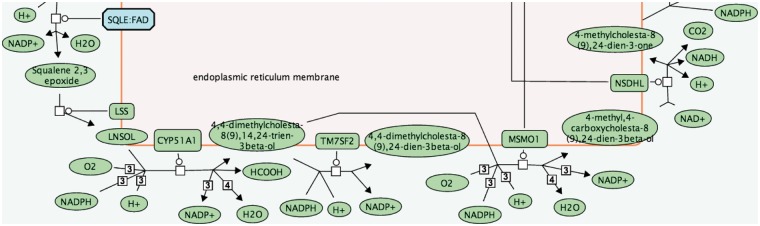
Pathway diagram segment using full small molecule names.

One benefit of these abbreviated names is a reduction in the space required to display small molecules in pathway diagrams. Figure 1 shows a segment of the cholesterol biosynthesis pathway labelled with abbreviated small molecule names; Figure 2 shows full names. The full names can be difficult to read and occupy a disproportionate amount of space in the diagram, making the flow of the pathway difficult to follow. The abbreviated names allow an improved layout.

This renaming process for small molecules in Reactome has been completed; as new small molecules are added to Reactome pathways they will be added to the table on the Reactome Wiki site. ChEBI has shown interest in incorporating the identified short names as synonyms and in using a similar process to find suitable short names for other chemicals. In principle, an abbreviation could be generated for any small molecule that participates in a biological pathway.

## Complex and set CV names

It is highly desirable to have names for sets and complexes that unambiguously describe their composition for searching and interpretation. The simplest option is to form a concatenated string from the names of the components or set members; this is the basis of the CV for complexes and sets. A colon is used as the separator between entities in complexes. A comma is used as the separator between entities in sets.

Reactome has more than one type of set. The simplest is the Defined Set, which contains molecules that are interchangeable functional equivalents. Reactome curation requires that members of a Defined Set must be experimentally verified functional equivalents; related ‘family members’ that are suggested to have the same function cannot be included. Candidate Sets are an extension of the Defined Set concept. Like Defined Sets, they include experimentally confirmed ‘members’, but also include annotated ‘candidates’, believed to represent additional members that have no direct experimental verification. To represent the distinction between Defined Sets and Candidate Sets, the CV names for candidate sets enclose candidates in round brackets, to distinguish them from members. This distinction may be unnecessary if the CV is used where there is no ‘candidate’ concept.

Homomeric multimers may use familiar terms such as dimer and tetramer to indicate the number of occurrences of the entity. If an entity occurs more than once in a heteromeric multimer, the name is preceded by the number of times it occurs and x, e.g. 2xPPOX:FAD is a complex of two molecules of PPOX and one of FAD.

### Examples of complex and set CV names

GRB2:SOS1—a complex of GRB2 and SOS1
IL3:IL3RA:IL3RB:JAK2—a complex of IL3, IL3RA, IL3RB and JAK2
RhoRac GEFs:GDP—a complex of Rho/Rac GEFs (a set) and GDP
IL3,IL3RA,CSF2RB—a set of IL3, IL3RA and CSF2RB
HRH2,HRH3,(HRH6,HRH8)—a candidate set of HRH2, HRH3, HRH6 and HRH8 where HRH6 and HRH8 are candidates.

### Representing hierarchical structure of complexes and sets in CV names

Complexes and sets may include complexes or sets as members. Recursive concatenation of the names of members defines the overall composition, but not the hierarchical structure. This might be considered a source of ambiguity, e.g. a complex ABC1:ABC2 might combine with another ABC3:ABC4 to form a complex ABC1:ABC2: ABC3: ABC4. A complex of the same name would be formed by combining a complex named ABC1:ABC2:ABC3 with an entity named ABC4. This ambiguity can be resolved by adding square brackets around pre-exisitng multimeric entities. The first example above would be named [ABC1:ABC2]:[ABC3:ABC4].

The addition of brackets to represent the limits of an existing complex or set can be continued as more entities are added. The complex above can be included as a component of a larger complex. A second set of brackets indicate its limits as a subcomplex within a larger complex:

[[ABC1:ABC2]:[ABC3:ABC4]]:XYZ1

The CV name can expand with the further addition of an entity:

[[[ABC1:ABC2]:[ABC3:ABC4]]:XYZ1]:MNO1

This can be expanded with the addition of a candidate set HRH2,HRH3,(HRH6)

[[[[ABC1:ABC2]:[ABC3:ABC4]]:XYZ1]:MNO1]:[HRH2, HRH3, (HRH6)]

This approach allows the unambiguous naming of any set or complex however large or complicated in terms of the identity of the entities that it includes, or the path that led to its existence.

Further examples of Reactome objects with names ‘before’ and ‘after’ applying the CV are available in Appendix I.

#### Names used to label complexes and sets in pathway diagrams

The CV name is formed from a concatenation of the names of components or members to exactly describe the content and composition of the named entity. This is ideal for a CV name where the length of the name is not a concern, but it can generate names that are too long to use as a convenient label in a diagram. This issue, separate from the initial task of creating the CV name, could be addressed with the following strategies to identify an alternative or shorter name that is a shortened form of the CV name.

When a set represents members of a protein family, the HUGO gene symbols will often have a common stem. This allows the use of the stem as a collective name, e.g. VAVs is sufficient to describe the family of VAV1, VAV2 and VAV3. This usage may be considered acceptable for use as a diagram label if the set is well defined and not anticipated to grow. It would not be considered acceptable for a partial set.

A variation on this approach can be used to represent sets containing some, but not all, members of a protein family, e.g. CCR1-5 can be used for a set containing CCR1, CCR2, CCR3, CCR4 and CCR5. CCR1,3–5 can be used for a set of CCR1, CCR3, CCR4, CCR5.

For more diverse set members, an alternative strategy is to use a name that describes their common function, e.g. CCR2 ligands, or VAV2-activated RhoGEFs. Here, the function, rather than common ancestry, is the property that the members of the set have in common.

Note that sets named using these strategies are always represented as a plural, e.g. a set of alcohol dehydrogenase complexes is named alcohol dehydrogenase complexes, not alcohol dehydrogenase or alcohol dehydrogenase complex, to avoid confusion with singular entities.

## Event (reaction) CV names

### Introduction

Like most other pathway databases, the names of steps in a Reactome pathway (reactions) are currently free text. Several terms may be used to name events that have similar mechanisms, e.g. binds, associates, forms a complex with, etc. This ambiguity makes it difficult to search for and identify all instances of an event subtype, e.g. the names used for binding events do not all contain the word bind, a text search for ‘phosphorylates’ does not identify all phosphorylation reactions. In addition, free-text reaction names make the editorial task of eliminating duplicate or near-identical reactions more difficult. To illustrate this, listed below are current examples of Reactome reaction names that are all phosphorylation events:

- Cyclin D:Cdk4/6 mediated phosphorylation of Rb and dissociation of phospho-Rb from the E2F1/2/3:DP-1 complexes
- ATP + beta-d-fructose => ADP + d-fructose 1-phosphate
- Phosphorylation of IRS
- Conversion of Glycerol to Glycerol-3-phosphate
- Activation of Cdc25C
- IRAK is activated

Some of these reaction names do not name the catalyst, some do not indicate that the described event involves phosphorylation. None of the names identifies the essentials of the event, namely the input molecules, the output molecules and the catalyst. An informed user might guess in most cases that these names describe a phosphorylation event. Using the Reactome website a user could refer to the molecular details, but a user searching Reactome for ‘phosphorylates’ would not discover any of the events shown above. Our aim is to remove the uncertainty and need for guesswork by providing CV names that facilitate successful and intuitive text searching.

The main advantage of a CV for reactions names is consistency; reaction names become easier for users to predict, search and understand. They also become useful for bulk computational queries and data mining. Further advantages are a reduction in Reactome curation effort, as reaction names can be automatically derived from the names of the entities involved, removing the need to carefully consider possible names and check them for consistency with existing similar reactions. Removing the free text nature of reaction name entry has the additional benefit of avoiding typographical errors or mismatches between the names of entities and their use as part of a reaction name, reducing the burden of editorial quality checking.

### Defining event categories

Event names can be standardized to a small set of CV terms that can be applied by following simple automatable rules. These terms, described below, are in many cases similar to or comparable with existing terms within existing ontologies and nomenclatures. Where multiple terms proposed here map to a single term or no available term in Systems Biology Ontology (SBO) or BioPAX we have proposed them as possible inclusions. To facilitate cross-referencing, we have constructed a mapping table, available here: 

https://docs.goo gle.com/spreadsheet/ccc?key= 0AnYqRv Z I4xked DJoR 1JP MX hqX 1RYRnVYemMyZmM4cWc& usp =drive_web# gid=7

The main advantage of this proposed CV for events is that the classes will be immediately familiar to most biologists, and offer improved granularity when compared with the terms used in comparable classifications.

In the following examples, capitalized text is used for emphasis and clarity only, the CV term is case-insensitive.

#### Transformation events

Transformation of entities is the default category for Reactome events, i.e. all reactions are considered to be transformations unless they can be assigned to another specified class of reaction. The reaction name has the style shown below:

a (,b and c) TRANSFORMS TO d (,e and f)

#### Binding events

If there are more input entities than output entities, the event is classed as a binding event. The test applied to determine binding can also be useful for verifying stoichiometric balance; at least one input entity should become a component of (one of) the output entities, or one of the input entities should include more entities as an output than it did as an input. The naming structure for binding events is shown below:

a BINDS b forming c

#### Dissociation events

If more output molecules are present than inputs, the event is a dissociation event. This can be tested by determining that at least one of the output entities is a component of an input entity or that an output complex reduces in size (to distinguish this class from certain transformation reactions). The naming structure for dissociation events is shown below:

c DISSOCIATES TO a AND b

#### Polymerization and depolymerization

Polymerization can be seen as a specialized form of a binding event. Polymerization events have their own reaction class in the Reactome data-model and are therefore easy to identify.

a (,b and c) POLYMERIZE TO x
x DEPOLYMERIZES TO a (,b and c)

#### Events with a molecular catalyst

Names for events with a catalyst specify the catalyst name (x). If the catalyst has an associated GO molecular function (GOMF), this may allow the use of a derived functional term, as explained below; otherwise, the name is structured as shown in this example:

x CATALYZES a (,b and c) TO d (,e and f)

In Reactome, all catalyst entities have an associated GOMF (1) term. The GOMF term can be used to derive or abstract a verb that can be used as part of the event name.

We have constructed a mapping table that defines the verb to be used for the 1463 GOMFs used in Reactome. The 1463 GOMF terms map to 61 verbs that can be used within the names of reaction events. In many instances, the selected verb corresponds to several GOMF terms. For example, GOMF terms containing the following words are all hydrolysis reactions:

- phosphodiesterase
- esterase
- lipase
- fumarylacetoacetase
- GTPase

This CV of 61 verbs constitutes a significant improvement in event categorization and naming consistency. It allows user text queries that are currently difficult or impossible. In addition, curators will spend less time deciding how to construct an event name as the appropriate verb and event name format will likely be available in the look-up table.

The current GOMF to verb table is available online (https://docs.google.com/a/ebi.ac.uk/spreadsheet/ccc?key = 0AnYqRvZI4xkedDJoR1JPMXhqX 1RYRnVYemMy Zm M4cWc&usp=sharing#gid=0). New GOMF to verb mappings will be added to this table as required.

The general format for reactions that have catalyst molecular functions defined in the lookup table is shown below:

Protein x ‘**verb**’ entity a (entities a, b, c) to x (,y and z)

##### Examples

- HLCS biotinylates ACACA
- PLB1 hydrolyses RPALM to atROL
- PTGDS isomerizes PGH2 to PGD2

#### Transferases

A separate look-up table has been created for transferase events, using GOMF terms to create a suitable verb but a slightly different structure in the CV name. In these events, a chemical group is transferred from a high-energy donor to a recipient, forming a covalently bonded modification. The CV name indicates the transfer of a specified molecular entity from the donor to the recipient. The round brackets indicate an optional extension that specifies the product:

Protein x **TRANSFERS** y **TO** a1 (**TO FORM** a2)

##### Examples

The GOMF term ‘alpha-1,2-mannosyltransferase activity’ is applicable to the human protein alpha-1,2-mannosyltransferase (ALG9). ALG9 mediates the transfer of a mannosyl moiety from the high-energy donor dolichyl phosphate d-mannose (DOLPM) to the growing *N*-glycan precursor used in the *N*-glycosylation of proteins. Italics here and in further examples below indicate an optional element. The CV name for this reaction is:

ALG9 transfers Man to (GlcNAc)2 (Man)6 (PP-Dol)1 (*to form (GlcNAc)2 (Man)7 (PP-Dol)1*)

Glycylpeptide *N*-tetradecanoyltransferases 1 and 2 (NMT1 and 2) mediate the transfer of the myristoyl (MYS) group from myristoyl-CoA to the N-terminal glycine (Gly) residue of transducin's alpha subunit (GNAT1). The CV name for this reaction is:

NMT1,2 transfer MYS to GNAT1 (*to form N-(C14:0)-GNAT1*)

#### Transport with no identified transporter or passive movement between compartments

Within Reactome pathways, the unassisted movement of an entity between cellular compartments is nonetheless regarded as an event. Translocation can be identified by comparing the compartment of molecular entities as inputs with their compartment as an output. If the entity is unchanged but associated with a different molecular compartment, this is a translocation event.

a **TRANSLOCATES FROM** [compartment x] **TO** [compartment y]

A similar situation occurs when entities move between cells. An example is cytosolic retinol moving from liver parenchymal cells to the cytosol of hepatic stellate cells for storage. In these circumstances, Reactome can use the ‘cellType’ attribute in the event name. This leads to a CV name in the following style:

a **TRANSLOCATES FROM** [compartment x of cell type 1] **TO** [compartment x of cell type 2]

#### Transport mediated by a known transporter

Transport that requires a transporter uses a different phrasing. The extra requirement is that there is a protein entity named as catalyst. The style for naming this class of events is shown below:

Protein x **TRANSPORTS** a (***FROM**** compartment x ****TO**** compartment y*)

#### Antiporter reactions

Here, different entities are exchanged in opposite directions across a membrane, mediated by an exchanger protein. The style that can be applied is shown below:

Protein x **EXCHANGES** a **FOR** b (*across the y membrane*)

An example is:

SLC9A9 **exchanges** Na+ **for** H+ (*across the late endosome membrane*)

#### Cotransporter reactions

Cotransporters (also called symporters) couple the movement of one or more entities with another entity(ies) in the same direction.

Protein x **COTRANSPORTS** a (b) **WITH** c (d)

SLC17A5 **cotransports** Neu5Ac **with** H+
SLC6A12 **cotransports** GABA **with** 3Na+ and 2Cl-

#### Activation

In Reactome, activation is used only to specify a conformational event that does not otherwise involve covalent modification or transport. Activation reactions can be identified as events where the input and output entities are the same with no change of compartment.

a is(are) **activated**

#### Regulation

Regulation, where an entity has a modulatory effect on an event, is represented using the style shown below:

a (positively or negatively) **REGULATES** x

## Applying CV names in Reactome

Generating and retrospectively applying a CV to an established database is a significant task, with technical and human usability issues to address. We have chosen to incorporate our CV in stages, rather than as a single step. We reason that the naming process for pathway objects should follow a ‘ground-up’ approach, where individual molecule names are defined and applied before the names of assemblies of molecules, i.e. complexes and sets, with renaming of reactions (pathway events) as the final step. At present we have completed the process of renaming peptides and small molecules. The CV is being used to direct current curation. Scripted processes will apply the CV terms retrospectively to existing entities and events to incorporate CV terms throughout the database. Ultimately, the process of generating CV names could be made part of Reactome’s curation or release processes so ensuring that new entities and events are automatically given CV names.
